# Analyzing antibacterial potential of bellamya *bengalensis* derived peptide-silver nanoparticle conjugates over surgical implant surfaces

**DOI:** 10.3389/fphar.2025.1658027

**Published:** 2026-02-25

**Authors:** Shafqat Qamer, Muhammad Shamim, Mahmoud H. El-Bidawy, Soha Abdallah Moursi, Mohd Saleem, Muhammad Aslam Siddiqui, Abrar Ali, Muhammad Shahid Iqbal

**Affiliations:** 1 Department of Basic Medical Sciences, College of Medicine, Prince Sattam Bin Abdulaziz University, Al-Kharj, Saudi Arabia; 2 Department of Surgery, College of Medicine, Prince Sattam bin Abdulaziz University, Al-Kharj, Saudi Arabia; 3 Department of Pathology, College of Medicine, University of Ha’il, Hail, Saudi Arabia; 4 Department of Ophthalmology, College of Medicine, University of Ha’il, Hail, Saudi Arabia; 5 Department of Clinical pharmacy, Prince Sattam Bin Abdulaziz University, Alkharj, Saudi Arabia

**Keywords:** arthroplasty, orthopedic implants, prosthetic joint infection, nanoparticles, AMP

## Abstract

**Introduction:**

Prosthetic Joint Infection (PJI) is a serious and potentially fatal consequence of total joint arthroplasty that is primarily due to the formation of bacterial biofilms on surgical orthopedic implants. Such orthopedic infections often face antibiotic resistance; therefore, novel antimicrobial substances are in demand to combat the biofilm-associated infections in orthopedic implants. This study investigated the potential of AgNPs-AMP conjugate coated implants in combating micro-organisms responsible for biofilm formation on orthopedic joint implants causing PJI.

**Methods:**

Green synthesized AgNPs from leaf extracts were conjugated with the AMP extracted from the Bellamya bengalensis, which was characterized using UV Spectroscopy, zeta potential. The antibacterial activity of the AgNPs, AMP and AgNPs-AMP conjugate were assessed using the Well diffusion method. The effect of biofilm formation and inhibition of Staphylococcus aureus, and Staphylococcus epidermidis on the ½ MIC, 1X MIC, and 2X MIC were investigated using agar diffusion test. In addition, MTT assay was performed to determine the cytotoxic effects of AMP-AgNPs conjugate on the L929 cell line.

**Results:**

The UV Vis spectroscopy and zeta potential confirmed the synthesis of AgNPs, as well as the conjugation of AMP with AgNPs. The AMP-AgNPs revealed their significant bactericidal potential against the S. aureus, and S. epidermidis, with the antibacterial testing. The quantitative and qualitative analysis of biofilm inhibition revealed that all the tested concentrations of AMP-AgNPs conjugate (½ MIC, 1X MIC, and 2X MIC) effectively inhibited the biofilm formation of S. aureus and S. epidermidis.

**Discussion:**

It was concluded that the treatment of different concentrations of AMP-AgNPs did not influence the cell viability of the L929 cells. This study demonstrated that the AgNPs-AMP can be coated with orthopedic implants to prevent biofilm formation on orthopedic joint implants.

## 1 Introduction

Joint replacement surgery has become a boon for patients with bone diseases by restoring joint function. There are several benefits of arthroplasty surgeries including good quality life, symptomatic relief, regaining physical activity, improving mobility, and regaining everyday routine freedom. There is a greater demand for orthopedic implants as more than 1.8 million fracture cases (Liu L. et al., 2013). And over 1 million knee and hip implants are performed annually in the United States (Etkin and Springer, 2017). A majority of surgeries are performed in osteoarthritis or rheumatoid arthritis cases over elderly patients who suffer from joint deterioration (Zhang et al., 2014). It is estimated that by 2050 the elderly population of 65 years or above will increase two-fold from 40.2 million in 2010 to 88.5 million in the United States alone (Vincent and Velkoff, 2010). The incidence of joint arthroplasties has significantly risen over the last several years on a global scale and it has benefitted worldwide patients with bone ailments (Cataldo et al., 2010). A successful joint replacement surgery reduces pain and discomfort, reclaims independence and physical activities, and enhances joint function and the patient’s life quality (Osmon et al., 2013a). Approximately, 800,000 joint arthroplasties are performed yearly in the United Kingdom and the United States, with estimates that the number will rise to more than 4 million by 2030 (Kapadia et al., 2016).

Biomaterials used in prosthetic medical devices for joint arthroplasties include metals, ceramics, composites, polymers, and natural materials and other foreign bodies that are extremely vulnerable to bacterial infections causing implant failure, out of which up to 15% of infection-associated hip implant failures require revision surgery (Khatoon et al., 2018; Zimmerli, 2014). Implant-Related Infections (IRIs) limit the efficacy of antibiotics, generate resistance, and allow infection to recur. IRIs may be divided into Prosthetic Joint Infection (PJIs) and Fracture-Related Infections (FRIs), both posing greater clinical and surgical challenges. Specifically, Prosthetic Joint Infection (PJI) has proven to be a very devastating postoperative consequence of implant failures (Lamagni, 2014). Being the most significant consequence of joint prosthesis, PJI occurs in 1.5–2.5 percent in terms of first treatments and even to 20 percent for further revision interventions. Moreover, the PJI is one of the most frequent causes (23%–25%) of revision surgery after knee arthroplasty, according to data from the United States and the United Kingdom (Osmon et al., 2013b). The PJI expenses are even up to 24 times more expensive including extended hospital stays, numerous surgeries, and extensive medical supplements (Osmon et al., 2013b). Among other factors that lead to orthopaedic implant failures are aseptic loosening and infection. Approximately, 18% of implant failures are attributable to aseptic loosening and 20% are related to implant-related infections (Raphel et al., 2016).

The primary factor causing implant-related infections or orthopaedic implant failure is biofilm formation on implants, which has been reported in patients who had undergone joint replacement surgery over 1–2 years (Kurtz et al., 2012). Among the most common biofilm formation is caused by the *Staphylococci* bacteria, whose one type, *Staphylococcus aureus,* is the most prevalent pathogen linked to total arthroplasty in the United States. Its other type, *Staphylococcus epidermidis,* is marginally more prevalent in Europe (Arciola et al., 2015; Montanaro et al., 2010). The *Staphylococci* can enter and remain inside host cells, enabling long-term retention of the microbes in bone by evading the immune system and antibiotic responses. As a result, bacteria form a biofilm, clustering into microorganisms and adhering to substrates and encasing in a sophisticated multicellular matrix (Aggarwal et al., 2013). Such complications need to be addressed on priority by finding out novel and effective measures to reduce such infections.

During the last 1 decade, several strategies and alternatives have been explored to resolve the issue of biomaterial formation in implant surgeries. These strategies have helped in modifying the surfaces of biomaterials, enhancing the delivery systems and creation of a novel and more effective antimicrobial substance. However, there is still a great need to initiate containment of implant-related infections and demonstrate an efficient antibacterial coating. Unfortunately, very limited technologies are available in the field of orthopedics and trauma that might offer a long-term solution.

Nanoparticles (NPs) are currently deemed to be an effective replacement for antibiotics which can potentially prevent infection on orthopedic implants (Sahoo et al., 2022). Metal-based nanoparticles, such as silver (AgNPs) and zinc oxide (ZnO NPs), particularly act as versatile tools to successfully resist bacteria. They disrupt bacterial cell membranes and generate ROS that damages bacterial deoxyribonucleic acid (DNA) and proteins, leading to cell death (Mondal et al., 2024; Gao et al., 2018). Thin-film coatings on medical devices, such as Ti_3_Au–Ag/Cu alloys, have demonstrated significant reductions in biofilm formation, offering a promising strategy to prevent infections. Antimicrobial peptides (AMPs), like murepavadin, disrupt bacterial membranes and show efficacy against multidrug-resistant strains, presenting a novel approach to combat antibiotic resistance. These innovations contribute to reducing infection risks and limiting the development of resistance in healthcare settings (Sahoo et al., 2022; Savvidou et al., 2020).

There are several other novel initiatives like silver nanoparticles coated exterior fixation pins, proximal femur or tibia mega-prostheses, and bone cement containing silver nanoparticles (AgNPs), that have revolutionized orthopedic science, in general and arthroplasty, in particular (Savvidou et al., 2020). All these novel inventions exhibit the characteristic inhibition of infections. Green synthesis of silver nanoparticles, for example, is an effective method to reduce the toxicity and enhanced activity using natural plant extracts for implants (Vincent and Velkoff, 2010; Arciola et al., 2015). Another method used for checking biofilm formation on implants is the use of natural antimicrobial peptides (AMPs), which are proteins or peptides that are the component of the innate immune response (Manobala, 2024). The AMPs are found in all forms of life like plants, animals, algae, fungi, archaea, and protists. These AMPs have been proven to be alternatives to diverse antibiotics that inhibit the growth of bacteria, viruses, fungi, and malignant cells (Brown and Hancock, 2006). Among the common AMPs, the mollusks are a diverse class of invertebrates that comprises snails, mussels, slugs, and octopuses, with which several members reside in marine and estuaries (Zhong et al., 2013). Thus, mollusks can be a valuable source of AMPs since they have intrinsic defenses to combat harmful pathogens (Zannella et al., 2017).

The study premised that a conjugation with NPs is beneficial to the treatment of infection because of their antimicrobial properties. It is argued in this study that AMPs prove to be a novel antibiotic alternative, which can be conjugated with biomaterial to predict an improvement in biomaterial performance. Conjugating AMPs with nanoparticles enhances the availability of the peptide at the targeting site. Such a conjugation either immobilizes AMPs or incorporate them into other material. Hence, when AgNPs are combined with AMP, their conjugation builds a potentially high synergy which leads to an effective therapeutic solution. Moreover, the conjugation of AMPs with nanoparticles also improves stability, antimicrobial potency, and also reduces toxicity (Gakiya-Teruya et al., 2020; Pal et al., 2016). This requires adoption of novel strategies to design peptides with enhanced antimicrobial activities (Pal et al., 2016). A need was thus felt to synthesize AgNPs at nanoscales and judge the ensuing enhanced efficacies, and devise a novel, stable, and potent AgNPs@AMPs agent that can stabilize and reduce the antibacterial potential over implant surfaces. Hence, the study premised that conjugation of Ag Nanoparticles and AMP can provide a more potent therapeutic approach for prosthetic joint infection (PJI) because of their synergistic action. Peptide-nanoparticle conjugates (PNCs) are a recently developed technology for biomedical purposes. The synergy between the two potential classes of substances enables greater regulation over their biological functions, circumventing each substance’s intrinsic constraints (Liu M. et al., 2013).

In this study, we have investigated the potential of AMP-AgNPs conjugate coated implants (Polypropylene, titanium, and Stainless steel) in combating micro-organisms responsible for causing Prosthetic Joint Infection. This occurs because alloys of certain characteristics such as superior mechanical form, resistance to fatigue and anti-corrosion properties, are preferred to metallic biomaterials over other materials such as polymers and ceramics to produce implants (Burduşel et al., 2018). A significant objective of this study, therefore, lies in finding alternative biomaterial coating agents, improvising that such novel agents should be used as a substitute to enhance the inherent organic properties of coating materials that can improve its processability and help in retaining an adequate level of bioactivity within tissues (Shekhawat et al., 2021). In order to accommodate these novel coating materials, it is important to ascertain how implants should have a design that can fabricate an implant in accordance with the choice of potential biomaterial. The study aimed at finding the best biosynthesized silver nanoparticle conjugates for the use in joint replacement. It also examined such potent antimicrobial agents (AMP-Np conjugates) for the coating on joint replacement biomaterials that can inhibit the biofilm formation and prevent the PJI. Specifically, the study framed the following objectives:

1. To synthesize Ag nanoparticles by biosynthesis methods and determine its antibacterial efficacy against *S. aureus* and *Staphylococcus* epidermidis.
2. To conjugate the AMP with biosynthesized (BS) nanoparticles and determine its antibacterial efficacy against *S. aureus* and *Staphylococcus* epidermidis.

Due to longevity of life and increased rates of obesity, and accessibility to modern medical advancements, total knee arthroplasty has become a very common treatment. However, the greater is the rate of arthroplasties, the larger are the complications and number of failures and the greater is also the number of revision and repetitions of arthroplasties. The combination of AgNPs and AMPs to prepare the implant coatings would significantly enhance the antimicrobial properties of the implants and improve the success rate. It is hypothesized in this study that the AgNPs@AMPs will not only improve the functionality of the implant surface but will also enhance the state of fixation and monitor the bacterial resistance.

## 2 Materials and methods

### 2.1 Procurement of orthopedic implants

Titanium alloy, Stainless steel and Polypropylene used in orthopedic implants currently were investigated in this study. The materials were procured from Vishal Surgitech Pvt. Ltd., India. The materials were cut into small pieces using laser cut technology (2 × 2 cm) and utilized for the biofilm experiments.

### 2.2 Collection of Bellamya bengalensis

Freshwater snail *Bellamya bengalensis* was collected from nearby ponds and lakes. The snails were rinsed in deionized water, the shells were broken, the soft bodies were separated, and they were kept at 20 °C.

### 2.3 Extraction of antimicrobial peptides

The separated soft bodies were homogenized in a blender with a 10% ice-cold acetic acid solution (1:3 w/v), followed by centrifugation at 5,000x rpm for 30 min at 4 °C (Poyil et al., 2021). Soluble proteins were precipitated at 4 °C with ammonium sulfate [(NH_4_)_2_SO_4_] at 100% saturation and stirred continuously for 1 h. The obtained precipitate was centrifuged at 5,000x rpm for 30 min at 4 °C and the pellets were suspended in 10 mM Tris-HCl buffer (pH 7.0). Peptides were isolated and kept at 4 °C. A dialysis bag filled with the precipitates was placed on the dialysis buffer (50 mM Tris-Cl at pH 6.8 and 100 mM NaCl) and continual stirring was carried out overnight at 4 °C. The proteins were collected by centrifugation at 13,000 rpm for 20 mins at 4 °C. The resulting supernatant was collected and used for further analysis (Dehghanifar et al., 2019).

### 2.4 Purification of antimicrobial peptides

The purification of antimicrobial peptides was performed by Ion-exchange chromatography (Dehghanifar et al., 2019). The DEAE cellulose bed 4 cm thick was used as a chromatographic column, which was rinsed with ethanol before use. Tris-Hcl (20 mM) was prepared, and the pH was adjusted to 8.5. Then, 2 mL of elutes were collected after being run for 4 h. Sodium phosphate citrate buffer was utilized as an eluting buffer (pH-7.2). Elute collection was carried out in a beaker and the crude AMP was poured into the column without disturbing the bed and allowed to settle for 20 min.

### 2.5 Green synthesis of nanoparticles

Fresh and mature *Hemigraphis colorata* leaves were collected from Riyadh, Saudi Arabia. The leaves were harvested and washed four times with de-ionized water and air-dried at room temperature. The leaves were then coarsely chopped into small pieces and mixed with 100 mL deionized water, which was agitated for 20 min at 60 °C. After boiling, the leaf extract was cooled to room temperature and filtered, and obtained leaf broth was stored at 4 °C. Leaf broth (5 mL) was mixed with 45 mL of 0.01M AgNO3 aqueous solution and left to react at room temperature. The colour shift in the reaction mixture from colourless to dark brown at various time intervals demonstrates the formation of AgNPs. The pellets were collected after centrifugation and dried for 1 h in a hot air oven at 50 °C and stored in Eppendorf tubes for later analysis (Nooralabettu, 2014).

### 2.6 Conjugation of antimicrobial peptides (AMP) and biosynthesized AgNPs

The water content of the purified peptides was removed and the peptides were lyophilized. 100 mg of extracted antimicrobial peptides (AMP) were dispersed in 1 mL of sterile deionized water and dropped into 100 mg of silver nanoparticles in a 1:1 ratio and sonicated for 5 mins allowing the AMP self-assembly on surface of Nanoparticles. The solution was incubated at room temperature for 30 min and filtered using 0.2 µM centrifugal filter (Pal et al., 2016).

### 2.7 Characterization of synthesized NPs and conjugate AMP-AgNPs

#### 2.7.1 UV Vis spectral analysis

UV–Visible spectroscopy (Shimadzu-UV 1800) was used to determine the absorbance wavelength of the biosynthesized AgNPs and AMP-AgNPs conjugates. A small aliquot of the test material was placed in a quartz cuvette and monitored for wavelength scanning between 350 and 500 nm. Sterile distilled water was used as a reference.

#### 2.7.2 Particle size and zeta-potential

The surface charge and also the average size and stability of biosynthesized nanoparticles were investigated using a Zeta analyzer (Malvern).

#### 2.7.3 Fourier transform infrared spectroscopy

FTIR analysis was used to determine the functional group present in the biosynthesized AgNPs and AMP-AgNPs conjugates. The tests were conducted out in KBr medium using a Thermo Nicolet Avatar 370 model FTIR spectrometer with a resolution of 4 cm-1 in the range of 400–4,000 cm-1. The test sample was mixed with KBr and a thin sample disc was produced. The emission spectra were collected using a Perkin Elmer LF-45 fluorescence spectrophotometer, and the existence of functional groups was analyzed.

### 2.8 Scanning electron microscope analysis

The sample suspensions were dropped onto a carbon-coated copper grid and it was allowed to dry completely under a lamp. The topographic analysis of biosynthesized AgNPs, AMP-AgNPs conjugate, and different implants coated with various concentrations of AMP-AgNPs conjugate was examined using a Field Emission-Scanning Electron Microscope (CARL ZEISS, Germany) (upto 66,000X) using a sufficient accelerating voltage (10 KV), vacuum (below 5 Pa), and magnification. Sputter coating was performed as the conducting material before analysis.

### 2.9 Transmission electron microscope analysis

The morphology and the size of the biosynthesized AgNPs and AMP-AgNPs were determined via TEM examination. TEM (HITACHI H-800) tests were performed at 200 kV. A drop of homogenized sample solution was placed on a carbon-coated copper grid and dried under a lamp to create the TEM grid. The size and thickness of the test samples were measured.

### 2.10 Anti-bacterial activity

#### 2.10.1 *In vitro* susceptibility test well diffusion method

Well diffusion method was utilized to determine the antibacterial effectiveness of biosynthesized AgNPs @AMP against two test organisms: *S. aureus* (ATCC® 25,923) and S. epidermidis (ATCC® 51,625). MHA (Muller-Hinton Agar) (Hi media, Mumbai) was prepared and sterilized before pouring into plates. Test microorganisms were grown in NB at 26 °C for 24 h with shaking at 150 rpm. About 300 µL 0.5 McFarland (106 Cfu/mL) 0.1 percent of each test organism’s culture solution was streaked across the Petri plate using a sterile cotton swab at a 60° angle for each streaking. On the agar surface of each MHA plate, a 6 mm well borer was utilized to bore wells. In each well, different concentrations of biosynthesized AgNPs@AMPs conjugates (1X, 40 μg mL-12X, 80 µg mL-1 3 × 120 µg mL-1) were loaded, and the plates were incubated at 37 °C for 48 h. Ampicillin (Amp) discs (256 μg/mL) was used as a positive control. In all the plates after 48 h of incubation, any antibacterial activity was measured with regard to inhibitory zones, which were observed and measured in millimetre (mm) by a Vernier calliper and the results were verified by two independent observers. In addition, the agar diffusion tests for all samples were also performed in triplicate, and mean results were taken (Khan et al., 2018).

### 2.11 Anti-biofilm activity

*Staphylococcus aureus* (ATCC no) and *S. epidermidis* (ATCC no) were grown in Mueller-Hinton broth and the ability to form biofilm was studied using the adherence assay on 96-well tissue culture. The 100 µL overnight bacterial suspension was added to each well in the microtiter plate, followed by the addition of ½ MIC, 1X MIC, and 2X MIC concentrations of AMP-AgNPs conjugate. Each strain was tested in triplicate. Wells with sterile Mueller-Hinton broth alone served as negative controls. *Staphylococcus aureus* and *S. epidermidis* were used as the positive control. The plates were incubated at 37 °C for 24 hrs.

After the incubation, the culture was withdrawn, and the plates were washed three times with 200 mL of phosphate-buffered saline it was dried in an inverted position in order to remove non-adherent cells. Ethanol (95%) was used to fix the adherent biofilm, it was stained using 1% crystal violet for 5 min. Then, the wells were washed using sterile deionized water to remove the unbound crystal violet and the plate was air dried for 30mins. The optical density was determined at the wavelength of 570 nm for each well (Jahangirian et al., 2013).

- Coating of Ag-AMP composites on the orthopaedic implants

Ag-AMP composites were coated on orthopaedic implants on slight modifications of methods described by Goda et al. (2022), Noumi et al. (2017). Implants were cut into small pieces and sterilized by autoclaving at 121 °C. Implants were dipped into a coating solution of a mixture of Ag-AMP conjugates (1X MIC and 2X MIC) of molten polyethylene glycol at stirring conditions. The mixture was heated at the range of 60 °C–70 °C in a water bath to obtain a homogeneous slurry. Implants were immersed in the slurry for 15mins and dried overnight at room temperature on a sterile surface under the air of laminar flow. The pieces were washed using sterile distilled water to remove loosely attached coating materials from the surface of the coated segments. The pieces were then left to air dry under an aseptic condition in laminar flow. SEM examination (CARL ZEISS, Germany) was performed to analyse the morphology of AMP-AgNPs conjugate-coated implants.

- Inhibition of Biofilm formation on coated implants using CLSM

Biofilm forming ability of *S. aureus* (ATCC no) and *S. epidermidis* (ATCC no) on the uncoated as well as 1X MIC, and 2X MIC of AMP-AgNPs conjugate coated implants were examined. The uncoated, as well as implants coated 1X MIC, and 2X MIC of AMP-AgNPs conjugate, were placed in the well of 6 well plates containing were inoculated with McFarland standard solution of bacterial suspensions were placed into each strip and incubated at 37 °C for 24 h (Jahangirian et al., 2013).

After 24 h incubation, the 6 well plates were gently removed, and rinsed by immersing them in PBS. Afterward, the bacterial biofilms were stained using the SYTO 9 bacterial viability kit. SYTO 9 was first diluted with PBS (1:1,000) before being introduced to each well in 1 mL of solution for 20 min at room temperature. Confocal laser scanning microscopy (CLSM) (LSM 710, Carl Zeiss, Germany) was used to analyse the morphology of the biofilm (Goda et al., 2022).

### 2.12 *Ex-vivo* RBC hemolysis assay

The blood samples were collected from healthy volunteers and were centrifuged for 5 min at 5,000 rpm. Then, the blood pellet was cleaned three times in a sterile phosphate buffer saline solution (pH 7.2) (Cerca et al., 2005). Different concentrations of AMP-AgNPs (1/2 MIC, 1X MIC, and 2X MIC) were added to the RBC suspension, and the reaction mixture was incubated at 37 °C for 60mins. Then, it was centrifuged at 1,500 rpm for 10 mins and the absorbance of the resultant supernatant was measured at the wavelength of 540 nm using a UV-Vis spectrophotometer. The PBS was used as negative control, and the 0.1% (v/v) Triton X-100 as positive control (Shabbir et al., 2013).

The hemolysis percentage was calculated using the following formula.

$$\text{Hemolysis }\%=\frac{\left(Absorbance\;of\;sample-Absorbance\;of\;negative\;control\right)}{\text{Absorbance}\;\text{of}\;\text{positive}\;\text{control}}\times 100$$

- *In-vitro* cytotoxicity analysis using MTT assay

The MTT assay was used to analyze the cytotoxicity of AMP-AgNPs against the L929 (normal fibroblast) cell line, which was purchased from the National Centre for Cell Sciences (NCCS), located in Pune, India. The cell line was cultured in MEM medium supplemented with fetal bovine serum and incubated at 37 °C with 5% CO_2_. After the initial 24-h incubation period, the growth medium was replaced with a freshly prepared growth medium containing varying aliquots of AMP-AgNPs. Then, 10 µL of MTT (5 mg/mL in PBS) was added to each well, and the plates were kept for further 4 h of incubation. The supernatant was collected, and 200 µL of DMSO was thoroughly mixed to dissolve the insoluble formazan crystals. The optical density was measured using a microplate reader at 550 nm (Tabassum et al., 2022). Then, the L929 cells treated with different concentrations of AMP-AgNPs conjugate was evaluated by acridine orange staining (Elsayed et al., 2015). The percentages of viability (%V) and inhibition (%I) were calculated using the following formula:

$$\frac{\text{Percentage}\;\text{of}\;\text{viability}\;\left(\%V\right)=100\;\left(A_{t}\;/A_{c}\right)}{\text{Percentage}\;\text{of}\;\text{inhibition}\;\left(\%I\right)=100\;\left[1-\left(A_{t}{\;/A}_{c}\right)\right]}$$

Where, A_t_–absorbance of treated cells; A_c_ - absorbance of control cells.

## 3 Results

### 3.1 Experimental summary

An overview of the experimental summary is provided in Table 1.

**TABLE 1:** Experimental summary.

Objective 1	Objective 2
Characterization of synthesized NPs and conjugate AMP-AgNPs	Evaluation of Anti-bacterial activity Well Diffusion Method: AgNPs@AMPs Synergistic combination
UV Vis spectral analysis	Biofilm Formation and Inhibition Assay
Particle size and Zeta-potential	Confocal Laser Scanning microscopy (CLSM)
Fourier Transform Infrared Spectroscopy (FTIR)	*Ex-vivo* RBC hemolysis assay
Scanning Electron microscope analysis (SEM)	*In-vitro* cytotoxicity assay (MTT assay)
Transmission electron microscope analysis (TEM)	

#### 3.1.1 Characterization of synthesized NPs and conjugate AMP-AgNPs

- UV Vis spectral analysis

**FIGURE 1:**
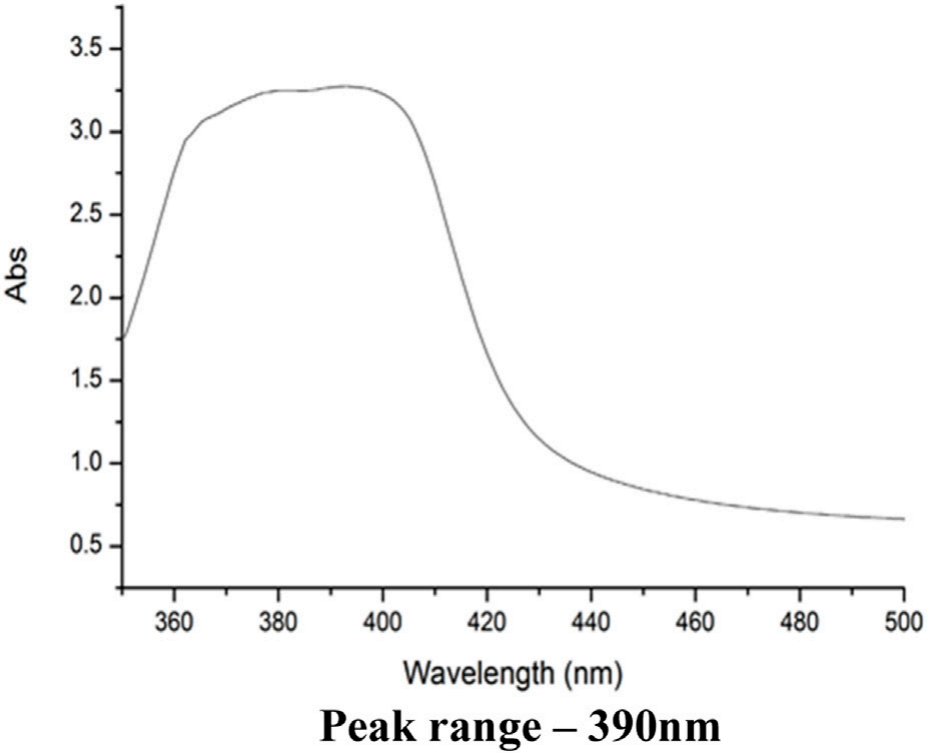
UV-Vis Spectrum for Ag-AMP conjugates.

Biosynthesized silver nanoparticles (AgNPs) using *Hemigraphis colorata* leaf extract exhibited a surface Plasmon resonance (SPR) peak at 400 nm, confirming successful synthesis within the typical SPR range for AgNPs (390–450 nm). Conjugation with antimicrobial peptides (AMPs) resulted in a slight redshift to 390 nm (Figure 1) and reduced absorbance intensity, indicating surface modification and successful AMP attachment.

- Particle size and Zeta-potential

Dynamic Light Scattering (DLS) analysis on the biosynthesized AgNPs and AMP-AgNPs conjugate displayed the mean particle size of 51.4nm and 77.1 nm respectively. The stability of the biosynthesized AMP-AgNPs was evaluated by measuring the zeta potential. The biosynthesized AMP-AgNPs exhibited −28.1 mV (Table 2).

**TABLE 2:** Stability and Particle size analysis of biosynthesized Ag-NPs@AMPs.

Sample	Average size (nm)	Zeta potential (mV)	PDI (Polydispersity index)
Ag-NPs@AMPs conjugate	77.1 ± 34.00	−28.1	0.621

**FIGURE 2:**
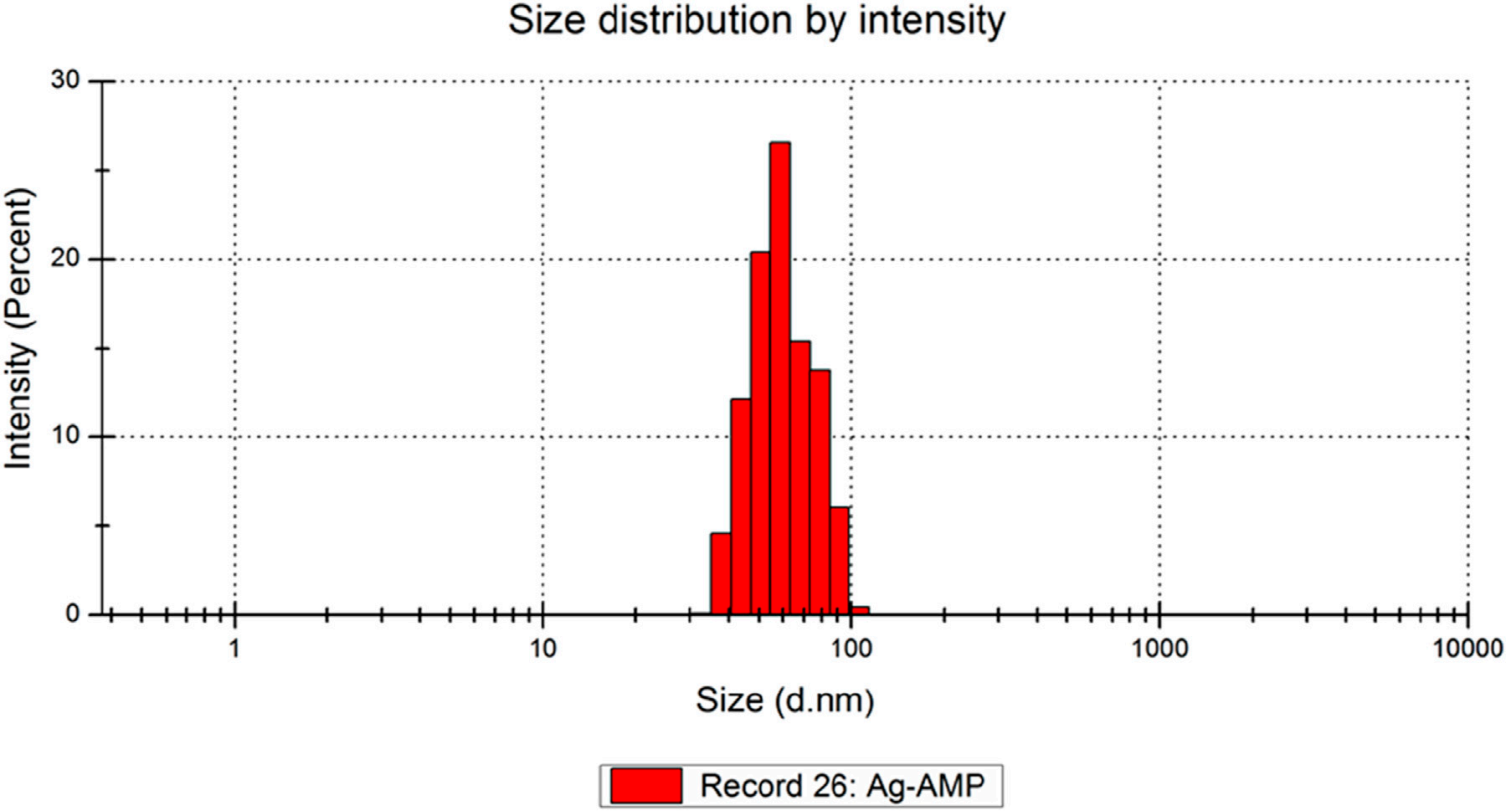
Size distribution Ag@AMP conjugates. Average size = 77.1 ± 34.00 nm.

**FIGURE 3:**
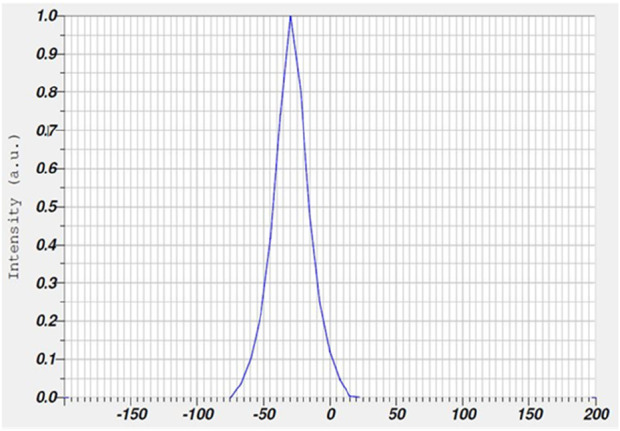
Zeta potential analysis for AMP-AgNPs.

The conjugation of AgNPs with AMP leads to an increase in the average size of the AMP-AgNPs conjugate to the biosynthesized AgNPs (Figure 2). As the net negative surface charges, which indicated the great stability of biosynthesized AgNPs (Figure 3).

#### 3.1.2 Fourier transform infrared spectroscopy

**FIGURE 4:**
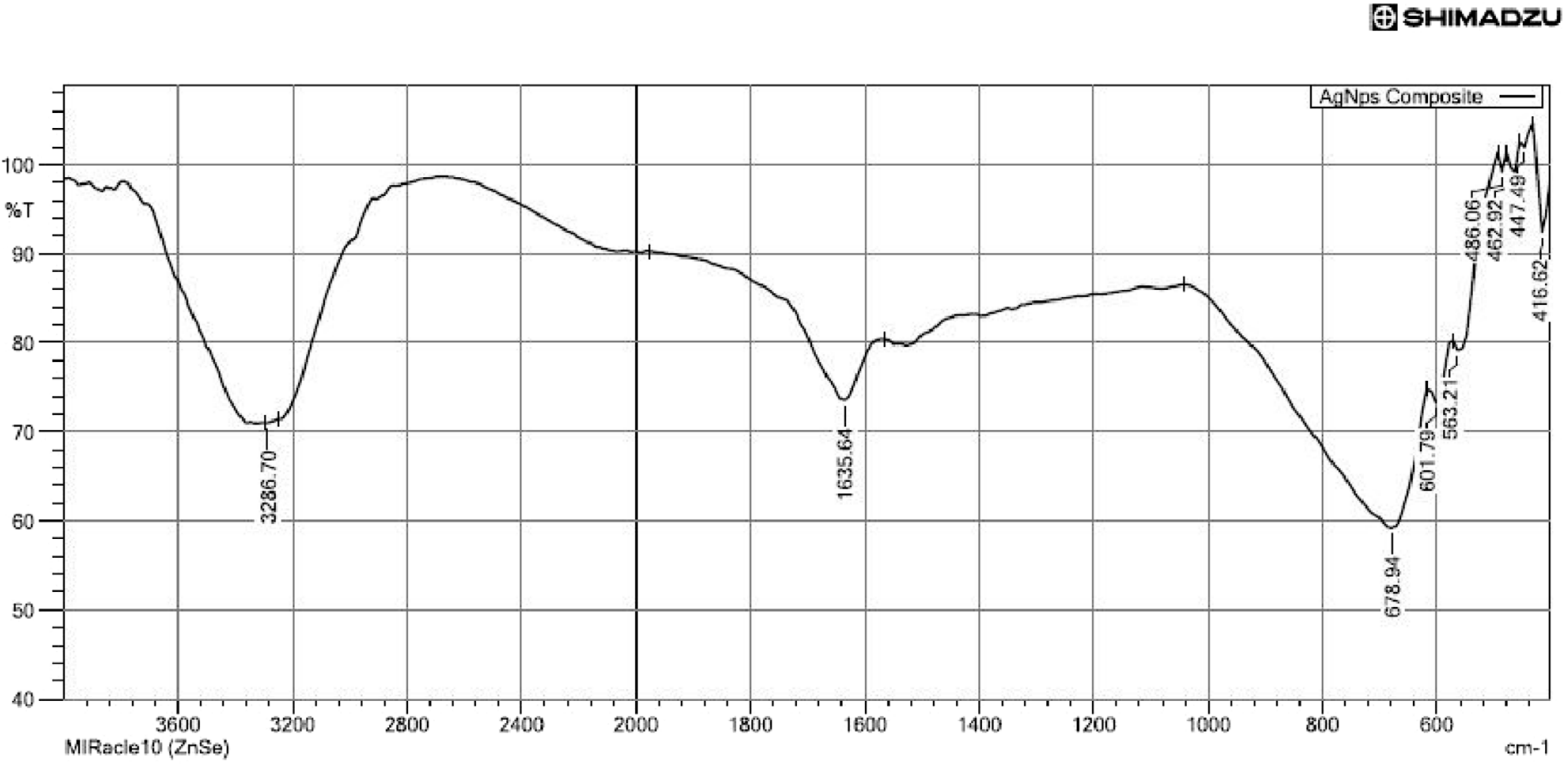
FTIR spectrum of AMP-AgNPs conjugate.

The FTIR spectra of AgNPs and AMP-AgNPs conjugates show similar functional groups with slight shifts in the peaks (Figure 4). Notably, the alkyl halide bands shifted from 671.23 cm to 1 in AgNPs to 678.94 cm-1 in the conjugate, and the C-H stretching band moved from 3309.85 cm to 1 to 3286.09 cm-1. These changes confirm the successful conjugation of AgNPs with AMP.

#### 3.1.3 Scanning electron microscope analysis

**FIGURE 5:**
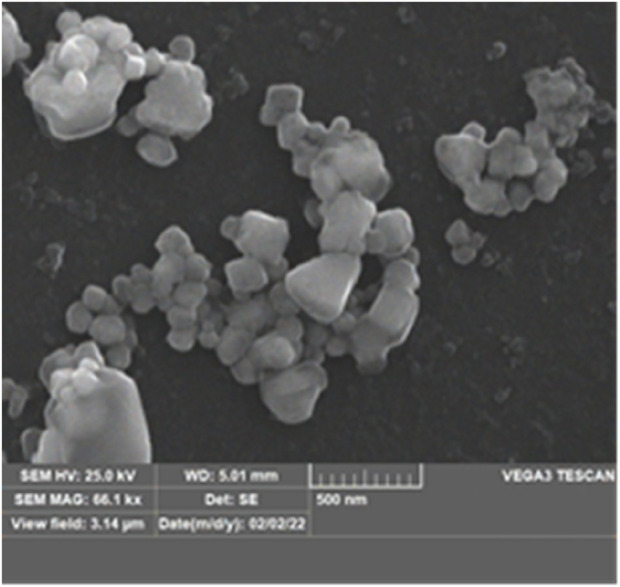
SEM image of AMP-AgNPs conjugate.

The SEM analysis showed that both AgNPs and AMP-AgNPs were spherical, with AgNPs being uniform in size AMP conjugation led to particle agglomeration and the formation of larger composites, confirming successful AMP attachment to AgNPs as shown in Figure 5. The entropic bonding of AMPs and AgNPs resulted in AMP-AgNPs conjugate showing the formation of composites at enhanced size. This confirmed the effective conjugation of AMP with AgNPs.

#### 3.1.4 Transmission electron microscope analysis

**FIGURE 6:**
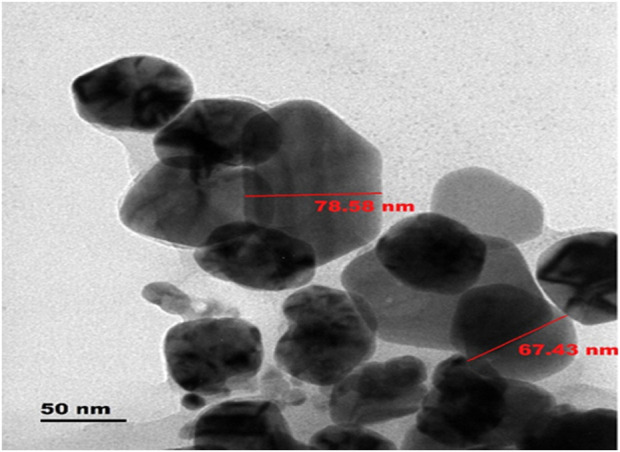
TEM analysis of AgNPs@AMPs (Sizes ranging from 67.43 to 90 nm) at scale bars: 50 and 100 nm.

TEM analysis revealed that both AgNPs and AMP-AgNPs were spherical or elliptical with smooth surfaces (Figure 6). AMP-AgNPs displayed particle aggregation, confirming successful conjugation. The average sizes were 54.76 nm for AgNPs and 67.43 nm for AMP-AgNPs, consistent with DLS results, with both under 100 nm in size (Figure 6).

#### 3.1.5 Well diffusion method: determination of the inhibition zone of AgNPs@AMPs

**FIGURE 7:**
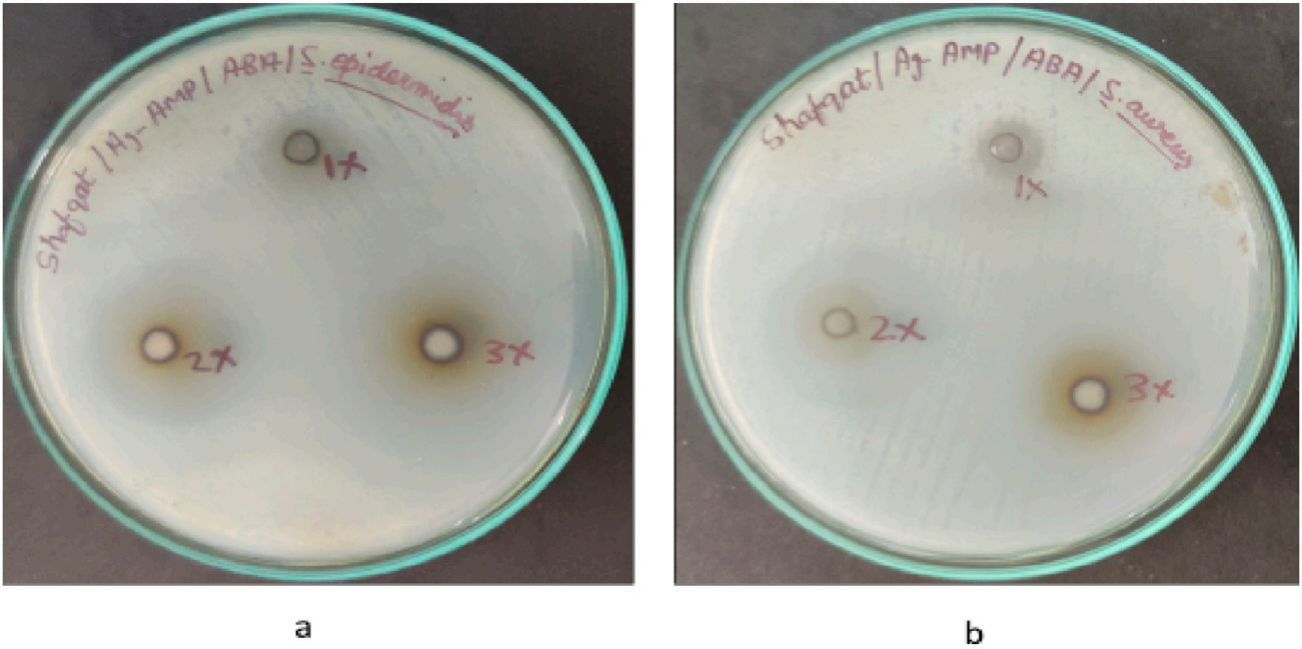
Visible clear zone produced by biosynthesized AgNPs@AMPs conjugates against. **(a)** *S. aureus* (ATCC® 25,923). **(b)** *S. epidermidis* (ATCC® 51,625)

**FIGURE 8:**
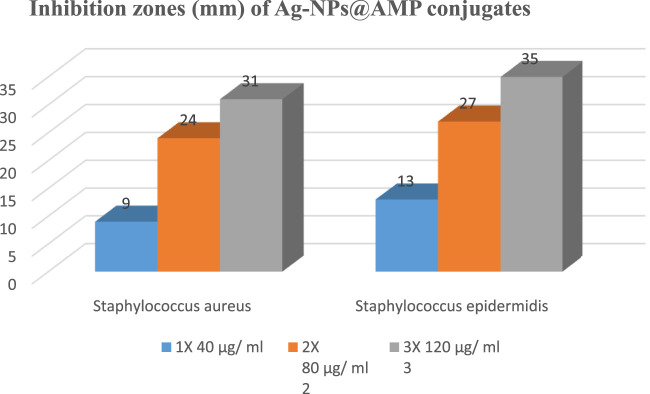
Antibacterial activity AgNPs@AMPs conjugates.

The antibacterial activity of AgNPs@AMPs was determined against two species of Gram-positive pathogens (Figures 7a,b; Table 3). Newly biosynthesized AgNPs@AMPs composite was subjected to antimicrobial testing. For the well 75 diffusion tests, the presence of clear zone around the AgNPs@AMPs disc suggesting that the AgNPs@AMPs possessed antibacterial activity which is able to inhibit the growth of Gram-positive cocci. The diameter of inhibition zone (IZ) for Gram-positive bacteria were ranged from 9 to 35 mm in different concentrations (1 × 40 μg/mL, 2 × 80 m μg/ml and 3 × 120 μg/mL). Ampicillin (Amp 256 μg/mL) disc was used as control against S. epidermidis, with an average inhibition zone (IZ) diameter of 35 mm followed by *S. aureus*, 31 mm (120 μg/mL) respectively as shown in Figures 7, 8.

**TABLE 3:** The diameter of zone inhibition (mm) of Ag-NPs@AMPs against *S. aureus* (ATCC^®^ 25,923) and *S. epidermidis* (ATCC^®^ 51,625).

S. No	Sample	Inhibition zones (mm)					
		*S. aureus*			*S. epidermidis*		
		1X	2X	3X	1X	2X	3X
1	AgAMP conjugate	09	24	31	13	27	35

Figures 7a,b and Figure 8 reveal the increasing trend of S. epidermidis zone of inhibition in comparison with *S. aureus* in the case of different concentrations of AgNPs@AMPs.

#### 3.1.6 Preparation and SEM analysis of AMP-AgNPs conjugate coated implants

**FIGURE 9:**
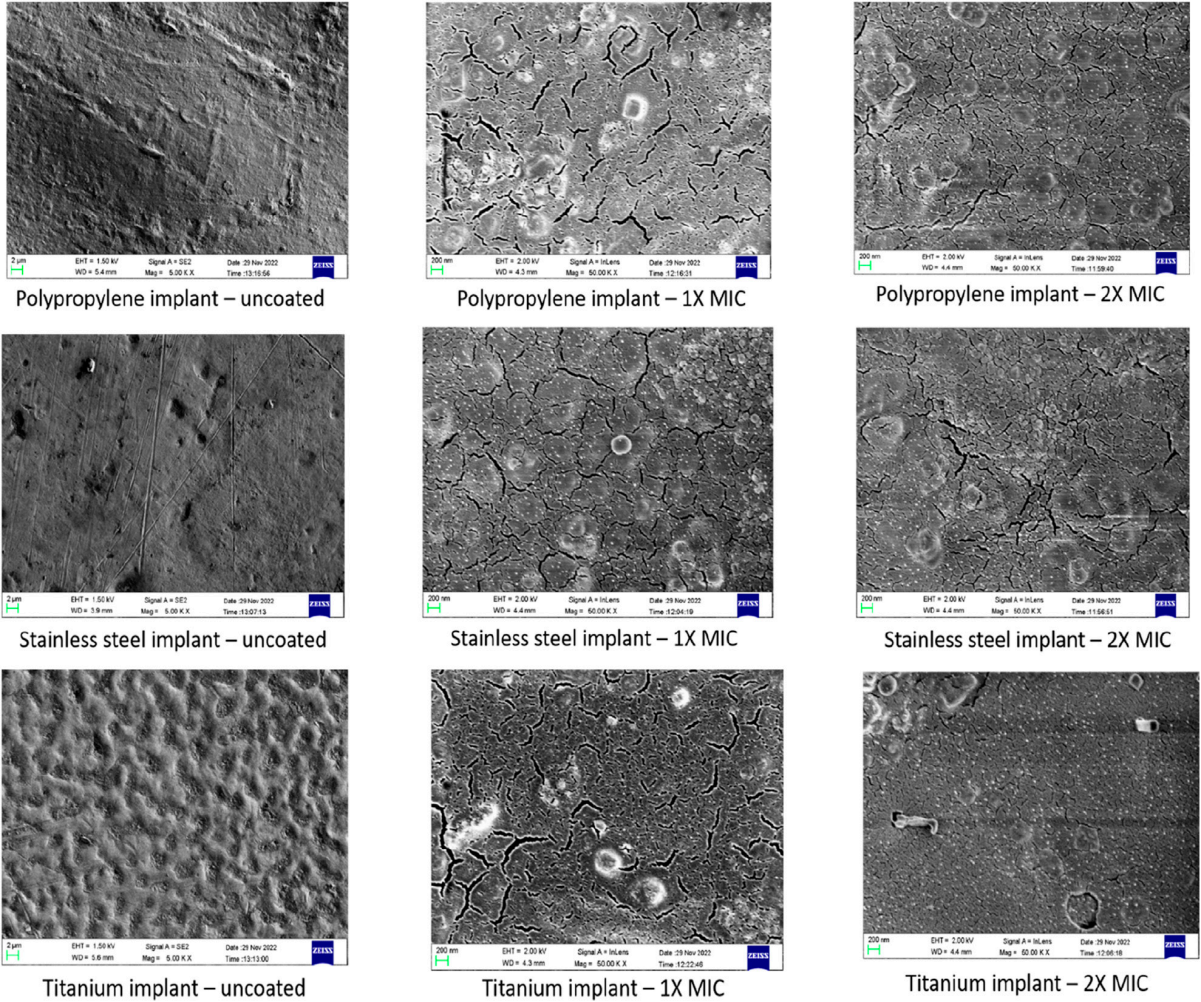
Microscopic examination of AMP-AgNPs conjugate coated implants.

Figure 9 depicts the SEM images of 1X, and 2X MIC of AMP-AgNPs conjugate coated implants. The AMP-AgNPs conjugate was observed to be evenly distributed on the surface of the polypropylene, titanium, and stainless-steel implants. The 2X MIC-coated implants were observed to have a thick and dense sheath-like surfaces.

#### 3.1.7 Confocal laser scanning microscopy (CLSM)

**FIGURE 10:**
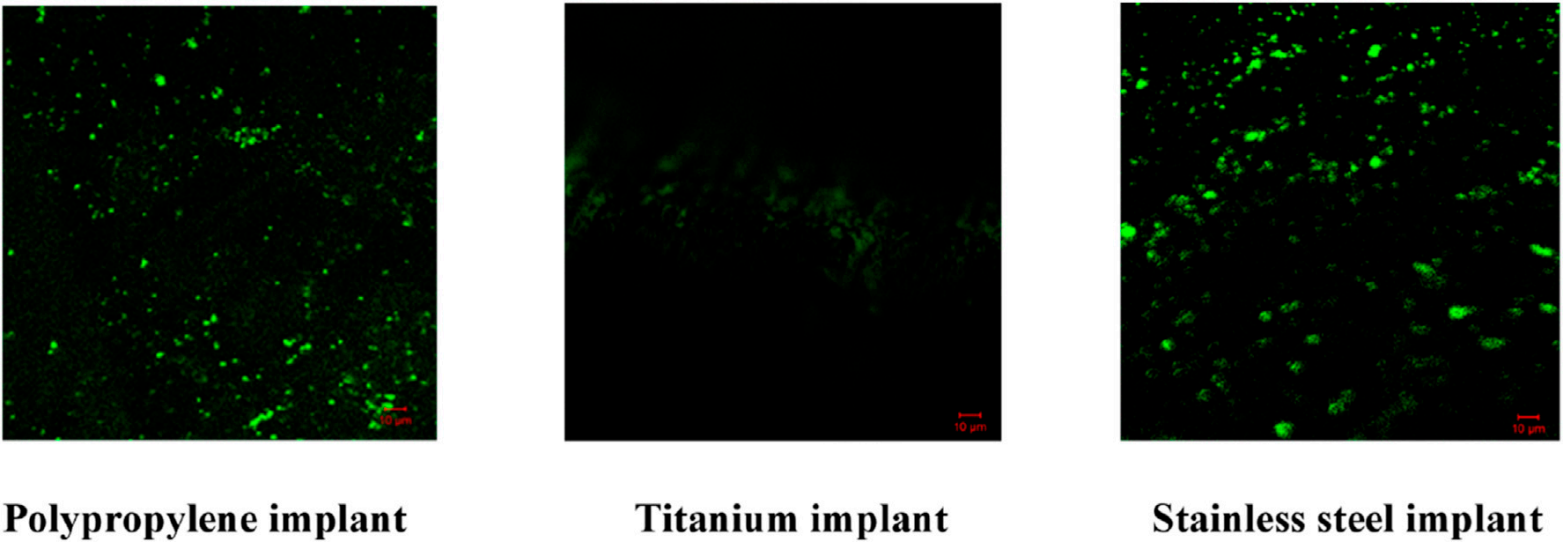
2D images of *S. aureus* biofilm inhibition of the AMP-AgNPs (1X MIC) coated implants.

**FIGURE 11:**
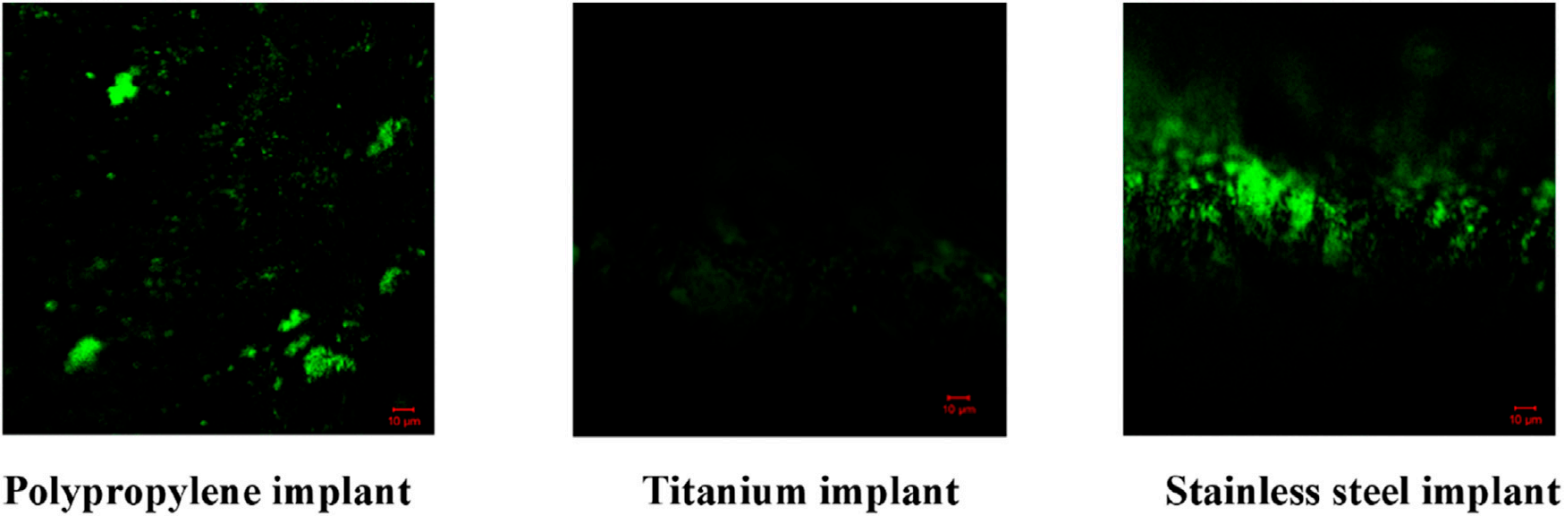
2D images of *S. epidermidis* biofilm inhibition of the AMP-AgNPs (1X MIC) coated implants.

**FIGURE 12:**
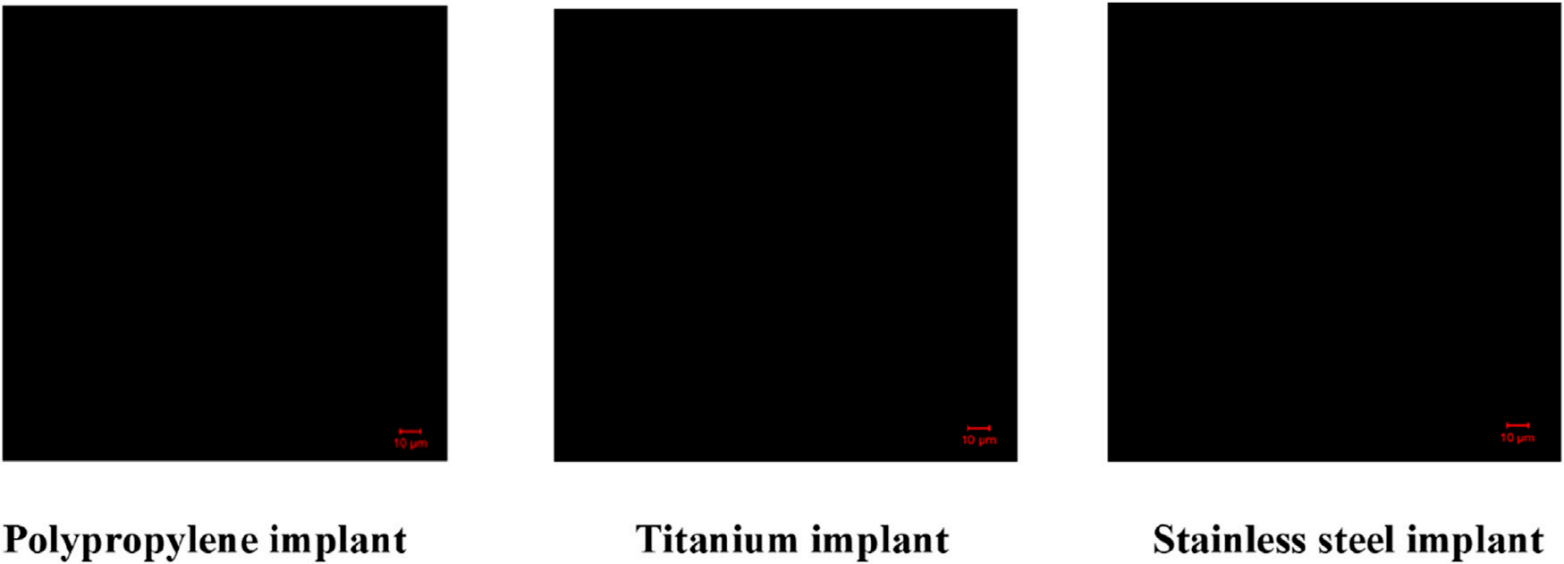
2D images of *S. aureus* biofilm inhibition images of the AMP-AgNPs (2X MIC) coated implants.

**FIGURE 13:**
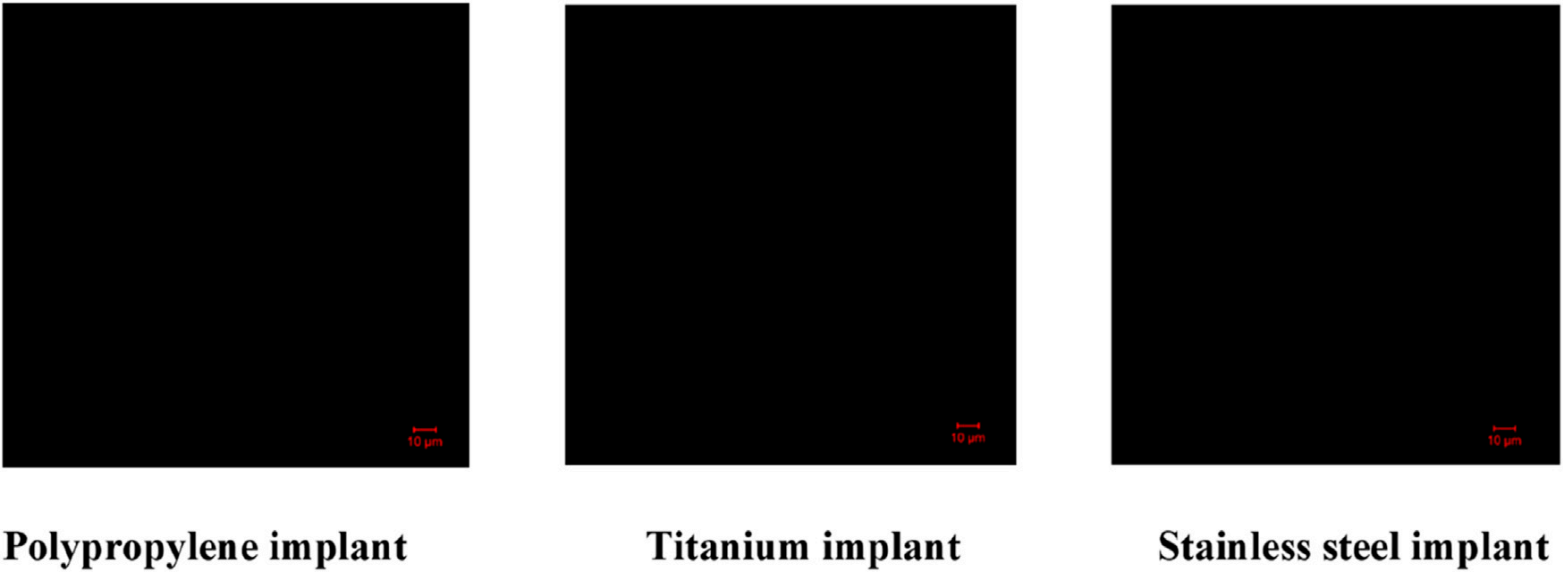
2D images of *S. epidermidis* biofilm inhibition of the AMP-AgNPs (2X MIC) coated implants.

CLSM analysis showed dense biofilms on uncoated implants, indicated by strong green fluorescence. AMP-AgNPs coatings reduced biofilm formation at 1× MIC and completely inhibited it at 2× MIC, with no green fluorescence observed (Figures 10, 11). The 3D imaging confirmed a significant reduction in biofilm volume, highlighting the strong antibacterial and anti-biofilm properties of the conjugates (Figures 12, 13).

#### 3.1.8 Evaluation of formation and inhibition of bacterial biofilm

- Biofilm formation

**FIGURE 14:**
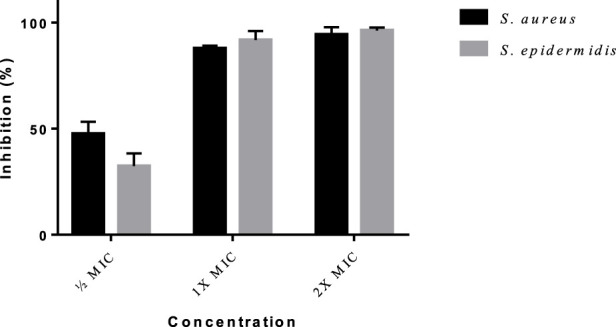
Inhibition of biofilm growth by different concentrations of AMP-AgNPs conjugate.

The state of biofilm growth (OD Value), when treated with different concentrations of AMP-AgNPs conjugates is shown in Figure Fig: 14. *Staphylococcus aureus* was observed to the high biofilm producer with the OD value of 0.46 ± 0.084 than the *Staphylococcus epidermidis* (OD value = 0.44 ± 0.004). All the tested concentrations of AMP-AgNPs conjugate (½ MIC, 1X MIC, and 2X MIC) effectively inhibited the biofilm formation of *S. aureus* and *S. epidermidis*.

The 2X MIC of AMP-AgNPs was able to decrease the biofilm formation significantly with the inhibition percentage of 94.2% ± 3.5%, and 96.2% ± 1.3% for *Staphylococcus aureus*, and *Staphylococcus epidermidis* respectively (Table 4).

- *Ex-vivo* RBC hemolysis assay

**TABLE 4:** Inhibition of Biofilm growth by the different concentrations of AMP-AgNPs conjugate.

Pathogens	Concentrations	Biofilm inhibition (%)
*S. aureus*	½ MIC	47.4 ± 5.7
	1X MIC	87.7 ± 1.2
	2X MIC	94.2 ± 3.5
*S. epidermidis*	½ MIC	32.3 ± 5.9
	1X MIC	91.7 ± 4.2
	2X MIC	96.2 ± 1.3

Values are represented in MEAN ± SD.

RBS hemolysis is used to evaluate the toxicity of the conjugates to human Red blood cells. The maximum percentage of hemolysis obtained was 6% ± 0.8% for 2X MIC of AMP-AgNPs, whereas, the 1X MIC AMP-AgNPs showed 4% ± 1.2%. Since the hemolysis were negligible and lesser than 10% AMP-AgNPs are considered to be safe and non-toxic to humans.

#### 3.1.9 *In vitro* cytotoxicity assay (MTT assay)

**FIGURE 15:**
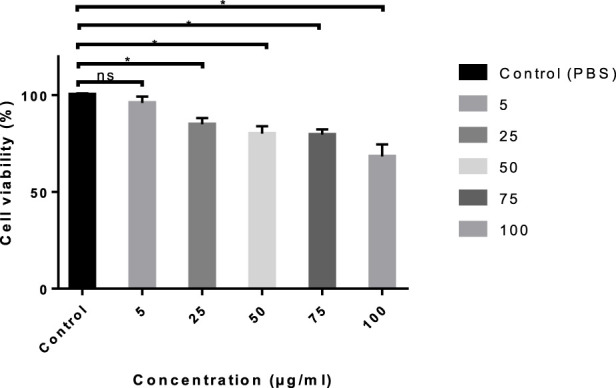
Cytotoxicity analysis of the AMP-AgNPs conjugate.

MTT assay showed that AMP-AgNPs conjugates were non-cytotoxic to L929 cells at 5 μg/mL, maintaining 96.3% ± 3.2% viability. ANOVA confirmed no significant difference from the control (p {{0.05). Acridine orange staining revealed strong green fluorescence, indicating healthy, viable cells (Figure 15). PBS is used as control (One way–ANOVA) to compare cell viability (%) with the control groups. Ns-non significant, * - p < 0.05.

## 4 Discussion

This study examined how AMP-AgNPs conjugate-coated implants can result in antibacterial synergism of NPs; with the highest level of such synergism was tested against *S. epidermidis*. Studies have reported the ability of AgNPs interaction of AMPs within their active groups (Shafagh et al., 2015; Naqvi et al., 2013), thus predicting a synergistic effect. Moreover, the AgNPs conjugates exhibited a more potent antibacterial effect against *P. aeruginosa* showing the same synergy when they interacted with different types of antibiotics after the conjugation of AgNPs to counter pathogenic microorganisms (Shahbandeh et al., 2021). It was also noted in this study that a higher synergy affected the NPs by enhancing the activity of AMP and cell penetration. The AgNPs and AMPs were less interactive but NPs alone facilitated the AMP penetration within the cell wall where the bactericidal action took place after the suppression of cell wall synthesis often ending with cell death (Masri et al., 2018). It was noted that the activity of AgNPs@AMPs was higher than that for biosynthesized AgNPs and AMP indicating the same trend against the tested microbes related to their large surface area and small size failing the easy penetration of the bacterial cell walls (Prabhu and Poulose, 2012).

In the end, a significant reduction of the antibacterial effect of the AgNPs@AMPs conjugate in relation to NPs activity was noted which requires. Further screening and tests in future studies.

This study thus accepted and validated the conjugation between biosynthesized AgNPs and AMP on bacteria. It also accepted the hypothesis that AgNPs@AMPs could induce only the membrane damage of bacteria; however, the pathways of AgNPs for the eradication of bacteria to treat bacterial infections still remain uncertain (Din et al., 2015). The silver nanoparticles (AgNPs) have been regarded as extremely promising for the development of new antimicrobials, which will provide a novel strategy for addressing a broad spectrum of bacterial infections. AgNPs can disrupt the bacterial cell wall, and inert the bacteria by attaching to its membrane proteins, and genetic material. Moreover, AgNPs can potentially interact with a multitude of enzymes, notably respiratory enzymes (López-Heras et al., 2015). The *Hemigraphis colorata* leaves-based AgNPs exhibited a maximum absorption peak at 415 nm, which is within the normal wavelength range of silver nanoparticles, i.e., 390–450 nm (Bethu et al., 2018), which corresponds with our findings.

The spherical-shaped nanoparticle with a size of 68 nm showed an absorption band at 418 nm (Reetha et al., 2020). The stability and surface charge of the AgNPs were examined using the zeta potential. Negatively charged surfaces help in maintaining the size and shape of nanoparticles by preventing them from aggregating and adhering together (Gakiya-Teruya et al., 2018). The surface area of the nanoparticles is one of the crucial factors determining antimicrobial potency. The AgNPs with the largest surface area illustrated the maximum release of Ag + against the bacterial cells, resulting in a significant bactericidal property (Paosen et al., 2017). These findings align with recent studies. For instance, a 2024 study by Nayak et al., demonstrated that PEG-grafted AgNPs formed stable assemblies, with short-chain PEG leading to dense, well-ordered films, while longer chains resulted in more complex structures (Zawadzka et al., 2014). Similarly, a 2024 study by Ogungbesan et al. reported that chitosan–silver nanocomposite films exhibited well-defined spherical AgNPs with uniform dispersion, as confirmed by SEM and TEM analyses (Nayak et al., 2025).

The potential interactions between AgNPs and the AMP conjugate as well as the functional groups found in the AgNPs were investigated using FTIR analysis. Similarly, the findings of (Ogungbesan et al., 2024). Reported the existence of a band at 1646.98 cm-1, which was determined to be the -NH2 stretch vibrations of amide. The broad peak at 3425.10 cm1 is made up of secondary amides. The current study reported that spherical-shaped nanoparticles with clear edges were observed in the SEM, and TEM images of AgNPs, whereas the addition of AMP leads to the aggregation of nanoparticles. The TEM analysis of AgNPs conjugated with antimicrobial Peptide Ubiquicidin (29–41) exhibited an increase in the average diameter as a result of peptide conjugation (Parasuraman et al., 2020), Morales-Avila et al., which is highly compromised with our findings.

AgNPs are typically utilized to transform the surface of orthopaedic implants in order to gain antibacterial and possible osteointegration properties (Morales-Avila et al., 2017). Retention of antibacterial activity for a long period of time to prevent the formation of biofilm is another crucial characteristic of implants. Biofilms are microbial populations that are adhered to a solid surface. Once the biofilm has developed on the implant surface, it prevents microorganisms from being treated with antibiotics and creates detrimental effect (Cheng et al., 2018). The MIC is commonly utilized as a significant indicator of antibacterial effectiveness. Our findings concluded that the extracted antimicrobial peptide from the Freshwater snail *Bellamya bengalensis* conjugated with biosynthesized AgNPs showed excellent antibacterial properties. Results from earlier studies suggested that conjugated peptides often kill microorganisms by rupturing the bacterial permeability barrier (Stoodley et al., 2002).

The conjugation of AMPs resulted in antibacterial synergism of NPs; the highest level of such synergism was against *S. epidermidis*. Studies have reported the ability of AgNPs interaction of AMPs within their active groups (Shafagh et al., 2015; Naqvi et al., 2013), thus predicting a synergistic effect. Moreover, the AgNPs conjugates exhibit a more potent antibacterial effect against *P. aeruginosa* (Shahbandeh et al., 2021), showing the same synergy when they interacted with different types of antibiotics after the conjugation of AgNPs to counter pathogenic microorganisms.

It was also noted in this study that a higher synergy affected the NPs by enhancing the activity of AMP and cell penetration. The AgNPs and AMP were less interactive but NPs alone facilitated the AMP penetration within the cell wall where the bactericidal action took place after the suppression of cell wall synthesis often ending with cell death (Masri et al., 2018). In the end, a significant reduction of the antibacterial effect of the Ag-NPs@AMPs conjugate in relation to NPs activity was noted which requires. Further screening and tests in future studies. A previous study done by (Hilpert et al., 2009), reported the significant anti-bacterial activity of the AgNPs synthesized using the aqueous extract of *H. colorata* leaves against the *Staphylococcus aureus*. Likewise, the AgNPs@AMPs complex exhibited higher anti-bacterial action against the *S. aureus* than the AgNPs or AMPs alone (Santhosh et al., 2018). AgNPs immobilized on the surface of titanium prevented bacterial adherence and icaAD transcription, which ultimately decreased the formation of bacterial biofilm. The immobilized AgNPs provide effective resistance against repeated bacterial attacks *in vitro*. Moreover, it lessens implant-related periprosthetic infection *in-vivo* (Zhou et al., 2023). The lowest MIC was recorded for the AgNPs-PEG-CIP, which demonstrated strong antibacterial activity with a considerable reduction in the MIC when compared to CIP and AgNPs alone (Qin et al., 2014). Likewise, the study of van (Ibraheem et al., 2022), Hengel et al., also disclosed the anti-biofilm activity of the AgNPs inserted into porous titanium implants against methicillin-resistant *S. aureus*.

SYTO9 dyes cannot cross undamaged membranes, however, they can attach to DNA when antimicrobial agents break the cell membrane and exhibit green fluorescence (van Hengel et al., 2017). The AMP-AgNPs coated in the implants disrupted the bacterial cell membrane and so intensified green fluorescence was observed in the microscopic investigation. A 2024 study by Lee et al. (2017). Reported that silver nanoparticles stabilized with C-pyocyanin significantly decreased biofilm formation in multidrug-resistant *Pseudomonas aeruginosa* and *S. aureus*, with CLSM images showing disrupted biofilm structures (Hussain et al., 2019). Similarly, a 2024 study by Manobala T et al. demonstrated that hydrogels containing biosynthesized AgNPs effectively inhibited biofilm formation by *S. aureus* and S. epidermidis, as evidenced by CLSM imaging (Chegini et al., 2024).

These studies corroborate our findings, highlighting the potent antibacterial and anti-biofilm properties of AMP-AgNPs conjugates. The biocompatibility of Nanoparticle Antimicrobial Peptide Conjugate with mammalian red cells and other nucleated cell types, primarily fibroblasts, osteoblast-like cell lines, and bone marrow-derived mesenchymal stem cells from human, mouse, rat, and rabbit, can be studied *in vitro* (Manobala, 2024). Red blood cells (erythrocytes) rupture during hemolysis, which is a sign that the red blood cells are being subjected to cytotoxic effects (Skerlavaj and Boix-Lemonche, 2023). The test samples are considered harmful to erythrocytes if there is more than 30% hemolysis (Velika and Kron, 2012), Table: 3 represents the RBC hemolysis activity of the AMP-AgNPs, which revealed that the 2x MIC of AMP-AgNPs exhibited a hemolysis percentage of 28.73 (less than 30%). Thus, it is considered to be safe, and non-toxic to humans. The limitation of hemcompatibility and enhanced mammalian cell toxicity were primary factors for the insignificant activity of silver-based antimicrobial coating on implants (Rasmussen et al., 2011).

## 5 Conclusion

Antimicrobial Peptides (AMPs) play a pivotal role in the innate immune system of invertebrates exhibiting a wide range of antimicrobial efficacy against bacteria, fungi, and viruses. AMPs have been viewed as prospective prospects for therapeutic purposes for the management of health ailments in humans, plants, and animals since the 1980s. AMPs have been regarded as a possible source for the development of new therapeutic compounds to treat infectious disorders for more than 30 years due to their considerable affinity for microorganisms and minimal toxicity for human cells. They are regarded as promising alternatives to traditional antibiotics to combat MDR bacterial infections (Ramstedt et al., 2009). The nanoparticles have a high promise for combating the problem associated with the multidrug resistance of bacteria (Manohar et al., 2019).

Nanoparticles conjugated with the antimicrobial agent can be used to lessen the toxicity of both substances toward human cells. Therefore, the combined antibacterial actions are boosted synergistically and the need for high dosages seems to be decreased (Lakkim et al., 2020). Hence, the use of nanoparticles is considered to be another effect. Active strategy for treating diseases caused by multidrug-resistant microorganisms. The antimicrobial properties of nanoparticles, particularly silver nanoparticles, are widely reported, and so conjugating them with AMPs has been suggested as a way to increase the activity (Allahverdiyev et al., 2011).

The current study demonstrated the potential of the AMP-AgNPs conjugate to inhibit the formation of biofilm by *S. aureus* and *S. epidermidis*. It showed how the antimicrobial activity of the AMP extracted from *B. bengalensis* can enhance significantly after the conjugation with AgNPs synthesized from *H. colorata*. Moreover, the 2X concentration of AMP-AgNPs does not exhibit any significant cytotoxic effects on the L929 cell line. However, it completely inhibited the biofilm formation of *S. aureus* and *S. epidermidis*. Hence, the AMP-AgNPs coated orthopedic implants will be the novel, non-toxic, and biocompatible approach for preventing Prosthetic Joint Infection.

The utilization of AgNPs as a pharmaceutical agent is restricted because of their susceptibility to cytotoxic effects against mammalian cells, despite their significant antibacterial properties and extensive pharmacological applications (Ramesh et al., 2016), But, our study demonstrated that the cytotoxicity of biosynthesized AgNPs conjugated with AMP against L929 cells was assessed using the MTT assay, which depends on the fact that viable cells convert MTT to purple formazan. As a result, the intensity of the color recorded at 550 nm was precisely related to the number of live cells. So, the AMP-AgNPs conjugate had no noticeable cytotoxic effects at 2X concentration.

Hence, the orthopedic implants could be modified with coatings with therapeutic substances to act as an antimicrobial defense, preventing infections from causing osteolysis and implant loosening. It is essential to prevent bacteria from adhering to the implant surface, to maintain a good interface between bone tissue and the substance, and to reduce the risk of infection while encouraging osseointegration (Mohanty et al., 2012). Last, but not the least, AMP-AgNPs have tremendous potential to be developed for commercial production. Future studies may determine what optimum conditions can be negotiated with AMP-AgNPs, and how the intensity of AgNPs can be increased within its limitations of small size and still retaining the stability. Such new studies may also examine any possible inhibitory mechanism of AgNPs in similar or different environment.

## Data Availability

The original contributions presented in the study are included in the article/supplementary material, further inquiries can be directed to the corresponding author.

## In memoriam

In memory of author Shafqat Qamer.
